# Preparation and Characterization of Polymer Membranes Impregnated with Carbon Nanotubes for Olive Mill Wastewater

**DOI:** 10.3390/polym14030457

**Published:** 2022-01-23

**Authors:** Muna A. Abu-Dalo, Maysa A. Al-Atoom, Mohannad T. Aljarrah, Borhan A. Albiss

**Affiliations:** 1 Department of Chemistry, Faculty of Science and Arts, Jordan University of Science and Technology, Irbid 22110, Jordan; maysaa_otoum@yahoo.com; 2 Department of Chemical Engineering, Faculty of Engineering, Jordan University of Science and Technology, Irbid 22110, Jordan; mtaljarrah@just.edu.jo; 3 Nanomaterials Laboratory, Department of Physics, Faculty of Science and Arts, Jordan University of Science and Technology, Irbid 22110, Jordan; baalbiss@just.edu.jo

**Keywords:** polymeric membrane, total phenolic compounds (TPC), carbon nanotube (CNTs)

## Abstract

In this study, polymer membrane(s) impregnated with carbon nanotubes (CNTs) were developed, characterized and evaluated for removing phenolic compounds from olive mill wastewater; thus, protecting the environment and public health. Polyethersulfone/functionalized, multi-walled carbon nanotube (PES/fCNTs) membranes were synthesized via the phase inversion method using PES and acid-treated CNTs. The prepared membranes were then characterized by scanning electron microscopy (SEM), Fourier-transform infrared spectroscopy (FTIR) and contact angle. Results obtained from this study indicate a more hydrophilic surface for the prepared PES/fCNTs membranes, with a higher pure water flux compared to the polyethersulfone (PES) membranes. In addition, the amount of fCNTs in the membranes was found to be the most significant factor affecting the morphology and water flux of the membranes. The PES/fCNTs membranes at 1 bar with 0 wt.% and 1 wt.% of CNTs showed water flux of 37.8 and 69.71 kg/h.m^2^, respectively. In addition, PES/fCNTs membranes with 0.5 wt.% fCNTs showed the highest total phenol content removal of 74%.

## 1. Introduction

Olive mill wastewater (OMW) is a black liquid effluent extracted from olive oil. In general, OMW is characterized as dark brown to black in terms of its color. It is acidic, with high organic matter content (total dissolved solids (TDS), chemical oxygen demand (COD), biochemical oxygen demand (BOD)) and a high concentration of phenols and potassium [1,2,3]. Phenolic compounds that are available in OMW are simple phenols and flavonoids; meanwhile, polyphenol results from the polymerization of the simple phenols. Similarly, the concentration range of phenolic compounds in OMW is from 0.5 to 24 g L^−1^ [3]. Globally, the OMW annual production is 30 × 10^6^ m^3^ [4], of which around 200,000 m^3^ is generated in Jordan [5,6]. Uncontrolled disposal of OMW creates a substantial environmental problem [7]. Phenol has a toxic effect on human health even in small concentrations [8]. Thus, it is necessary to remove phenols from olive mills before discharging it. Many studies focused on OMW treatment by several methods [9,10,11].

The current paper focuses on membrane technology which plays a significant part in the separation and treatment processes [12]. These technologies can be considered as simple, and as requiring compact space and low energy [13].

Commonly, polymeric materials, for instance, cellulose, polyethersulfone (PES), polysulfone (PSf) and polyamide, are used for the synthesis of organic membranes [14]. These materials are still widely used in wastewater treatment because of their unique porous structure, good mechanical characteristics and comparatively low-cost operation and processing. In addition, they are created through energy-saving techniques and are environmentally friendly [15,16]. Polyethersulfone (PES) is the most common polymer material used for membrane synthesis. It has high chemical resistance, polarity and flexibility due to its resonance structure [17,18]. Moreover, PES has a strong heat-aging resistance, high heat-distortion temperature and is more suitable for water processing compared with PSf [19].

In general, polymeric membranes are hydrophobic by nature (low water flux and serious membrane fouling) and have relatively low separation performance. However, hydrophilicity and separation performance can be enhanced by nanomaterials, such as carbon nanotubes (CNTs) [14,20], titanium oxide [21] and graphene oxide [22], to enhance separation performance, improve the hydrophilic properties and decrease the fouling problem.

Son et al. [20] observed that membrane hydrophilicity, average pore width, total pore area and porosity were improved after adding fCNTs into the PES support layer in thin-film composite (TFC) reverse osmosis (RO) membranes. In addition, Tang, Y et al. [23] presented the effects of a single-walled carbon nanotubes (SWCNTs) interlayer on the TFC membrane’s properties and performance. They found that a SWCNTs interlayer considerably improved water flux and the superior membrane structure parameters (S) value in the TFC membrane, leading to an increase in the internal concentration polarization (ICP).

Carbon nanomaterials (CNMs) are attractive materials because of their exceptional structures and electronic properties which make them the most used type for various applications. Their benefits for wastewater treatment are due to their large specific surface area, great capacity and affinity towards aromatics [24,25,26]. CNTs have large surface area and abundant porous structures. In recent years, many studies have been performed to investigate the effect of CNTs on polymeric membrane performance. For example, Celik et al. [27]. showed that adding multi-wall carbon nanotubes (MWCNTs) to a PES membrane improved the membrane properties such as roughness, porosity, flux, membrane separation properties and hydrophilicity. In another study by Wang et al. [28] a great improvement in fluxes and salt rejections was found for mixed matrix membranes (MMMs) compared to PES membranes. Moreover, Peydayesh et al. [29] showed that fabricating a positively charged, hybrid nanofiltration membrane using triethylenetetramine (TETA) functionalized MWCNTs into the PES matrix and enhanced the membranes’ hydrophilicity, dye rejection, antifouling property and thermal and mechanical stabilities compared to PES membranes. A work done by Vatanpour et al. [30] illustrated that using MWCNTs in a PES membrane increased hydrophilicity and water flux.

Daramola et al. [14]. observed the performance of a PSf membrane with a functionalized CNT and PVA coating. They found an increase in rejection with pressure decrease, and an increase in CNTs content for the uncoated PVA layer. They also found that the PSf membrane with 1 wt.% fCNTs showed rejection of about (99.9%) for kerosene and petrol and, for phenol, a rejection of 65%. However, PES membranes with CNTs have not been used in treating synthetic OMW samples until now.

This study aims to explore the feasibility of using PES impregnated with functionalized CNTs for the rejection of phenolic compounds from OMW. Membranes were prepared by phase inversion method and the effect of the CNTs in enhancing membrane hydrophilicity and membrane performance in removing phenolic compounds was tested. PESf/CNTs membranes, along with pure PES membranes, were characterized by scanning electron microscope (SEM), X-ray diffraction, contact angle test and FTIR spectroscopy.

## 2. Materials and Methods

### 2.1. Materials

Vanillic (4-hydroxy-3-methoxybenzoic) acid (+98.5%), caffeic (3,4 dihydroxycinnamic) acid (98%), *p*-Coumaric acid (ACROS Organics, Thermo Fisher Scientific, Fair Lawn, NJ, USA) (98%), gallic (3,4,5-trihydroxybenzoic) acid (99%) (Xilong Chemical Industry Incorporated Co., Ltd, Guangdong, China) and tyrosol ((2-(4-hydroxyphenyl) ethanol) (98%) (Aldrich, German) were used to create an OMW synthetic solution.

CNTs (NC7000, Nanocyl company, Sambreville, Belgium), polyethersulfone (PES) (MW = 58,000 g/moL with polymerization degree 259, Goodfellow Company, Huntingdon, London), polyvinylpyrrolidone (PVP) (MW = 58,000 g/moL, ACROS Organics, Thermo Fisher Scientific, Fair Lawn, NJ, USA), 70% *N*-methyl-2-pyrrolidinone (NMP) (SupraSolv, Darmstadt, Germany), Folin-Ciocalteu phenol reagent (Scharlau, Barcelona, Spain) and sodium carbonate anhydrous (Na_2_CO_3_) (Fischer, Shanghai, China) were also used in this study.

### 2.2. Synthesis of OMW

Synthetic OMW was prepared by mixing five phenolic acids that are normally present in real OMW. The percentage of each phenolic compound in the synthetic OMW was based on Esteves et al. [31] recipe as follows: 11.11% of vanillic (4-hydroxy-3-methoxybenzoic) acid, caffeic (3,4 dihydroxycinnamic) acid and gallic acid (3,4,5-trihydroxybenzoic acid) and 22.22% of *p*-Coumaric acid and 44.44% of tyrosol ((2-(4-hydroxyphenyl) ethanol) were dissolved in distilled water and mixed by sonication (SONICS viber-cell) for 15 min to ensure full dissolution.

### 2.3. Surface Modification of CNTs

To increase the dispersion of CNTs in organic solvents and to remove their impurities, the CNTs were prepared in a mixture of HNO_3_ and H_2_SO_4_ [32]. The approach was as follows: Firstly, 200 mL of 3:1 (*v*/*v*) H_2_SO_4_:HNO_3_ mixture was added to 1.0 g of CNTs and sonicated for one hour. Secondly, the mixture was refluxed at 60 °C for 6 h. The solution was diluted with DI water and left to cool down overnight. Finally, the functionalized CNTs (fCNTs) were washed and centrifuged (Centurion Scientific C2 series) up to reach a pH of approximately 5 and then dried overnight in an oven at 100 °C.

### 2.4. Membrane Preparation

The polyethersulfone PES/fCNTs membranes were prepared by phase inversion method. The casting solution contained 20% PES, 10% polyvinylpyrrolidone (PVP), 70% *N*-methyl-2-pyrrolidinone (NMP) and several amounts of fCNTs (weight ratios 0, 0.1, 0.5, 1% of the PES). Moreover, for fabrication of membranes, fCNTs were added to NMP and then sonicated for 1 h to ensure dispersion of fCNTs in solvent. The PES and PVP were then added to fCNTs/NMP mixture and stirred overnight. Next, the mixture was reserved overnight. The membrane solution was casted by doctor blade extrusion method in which membrane solution was poured onto glass plate and spread using the doctor blade by hand and then immersed into a de-ionized water bath for a few minutes until the membrane separated from the glass plate. The synthesized membranes were then washed with deionized (DI) water several times and kept in water prior to their use.

### 2.5. Membrane Characterization

For surface chemistry analysis, the CNTs were characterized by Fourier-transform infrared spectroscopy (FTIR) (Bruker-Avance) in the 400–4000 cm^−1^ wavenumber range. The crystallinity and phase’s identification X-Ray diffraction (XRD) (Rigaku Ultima IV, Rigaku Company, The Woodlands, TX, USA) were obtained using Cu-Kα radiation (λ = 1.542 Å).

The surface and cross-section morphology of membranes were directly observed by scanning electron microscope (SEM) (Quanta FEG 450, Amsterdam, The Netherlands). The membranes were dried and coated with gold sputter (Q150R Rotary-Pumped Sputter Coater/Carbon Coater, Quorum Technologies, Lewes, UK). The surface hydrophilicity was evaluated by drop shape analyzer using a contact angle goniometer (Attension Theta Lite, Biolin Scientific Company, Västra Frölunda, Sweden).

By the gravimetric method, the porosity was obtained, where membranes were weighted after removing the water (m1) and after drying (m2). Membrane porosity (*ε*) was determined according to Equation (1):

(1) $$\varepsilon =\frac{m1-m2/\rho_{H2O}}{\left(m1-m2/\rho_{H2}\right)+\left(m2/\rho_{\mathrm{PES}}\right)}$$

where ρ_H2O_ is the density of water, 1.0 g/cm^3^, and ρ_PES_ is the density of PES, 1.37 g/cm^3^ [33].

### 2.6. Membrane Performance

Membrane performance was examined by dead-end filtration system (Sterlitech, HP4750, Auburn, WA, USA), where the effective area was 14.6 cm^2^. The membrane at the beginning was cut and put in according to the size of the dead-end cell. After that, the membrane was pre-compacted at 1.5 bar for 30 min with feed solution (DI water). The permeate flux was tested for all membranes at 1 and 2 bars (ultrafiltration) for 30 min. All experiments were performed three times, then the average values were calculated.

The permeation flux (J_0_) is derived from the following Equation (2):

(2) $$\mathrm{flux}\left(J_{0}\right)=\frac{W}{A.t}$$

where *W* is the weight of filtrated solution (grams), *A* is the membrane surface area (meter cubic) and *t* is the experiment time (hour).

To determine the phenol rejection efficiency, a synthetic OMW with concentration around 75 ppm was prepared. The OMW was filtered through the system and the concentration of phenols was analyzed by UV/Vis spectrophotometer according to the Folin-Ciocalteu method at a wavelength of 750 nm. Phenol’s rejection efficiency was calculated using the measured feed and permeate concentrations following Equation (3):

(3) $$\mathrm{Rejection}\left(\%\right)=\frac{C_{f}-C_{p}}{C_{f}}*100\%$$

where C_f_ and C_p_ are the concentrations of the feed solution and permeate, respectively.

### 2.7. Determination of Total Phenolic Compounds

The total phenolic content was determined by using the Folin-Ciocalteu method as follows: Four standard solutions of gallic acid (25, 50, 75 and 100 μg/mL) were prepared. After that, 1 mL of each filtrated sample and standard was added to a 25 mL volumetric flask. A total of 9 mL of distilled water was added to the mixture, followed by 1 mL of Folin-Ciocalteu phenol reagent, then the mixture was shaken for 5 min. A total of 10 mL of 7% sodium carbonate anhydrous (Na_2_CO_3_) solution was added to the mixture, then more water was added to bring the volume to the mark on the flask. The mixture was incubated for 90 min at room temperature, and the absorbance was measured using UV-visible spectrophotometer (AE-UV1608, A&E Lab Company, London, UK). The amount of TPC was determined from the gallic acid calibration curve [34].

## 3. Results

### 3.1. Fourier-Transform Infrared Spectroscopy (FTIR)

**Figure 1:**
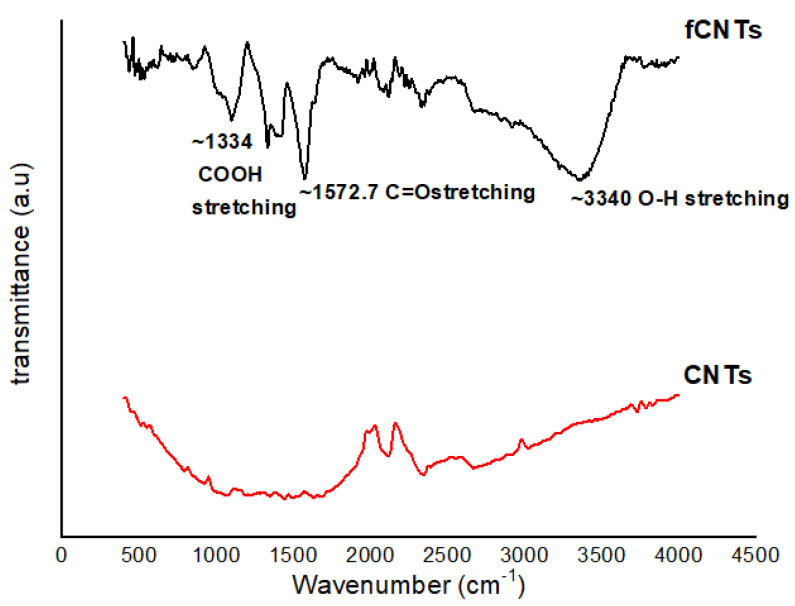
FTIR spectra of CNTs and functionalized CNTs.

FTIR was used to determine the new functional groups onto the CNTs surface after acid treatment. The FTIR spectra of modified CNTs is shown in Figure 1. The new peaks appeared at 1334, 1572.7 and 3345 cm^−1^ after surface modification of CNTs and are related to COOH, O–H and C=O bonds, respectively, for the attachment of carboxyl, alcohol and carbonyl functional groups onto the CNTs surface [27]. These groups enhanced the hydrophilic properties of CNTs and increased the dispensability of oxidized CNTs in aqueous solution [30].

**Figure 2:**
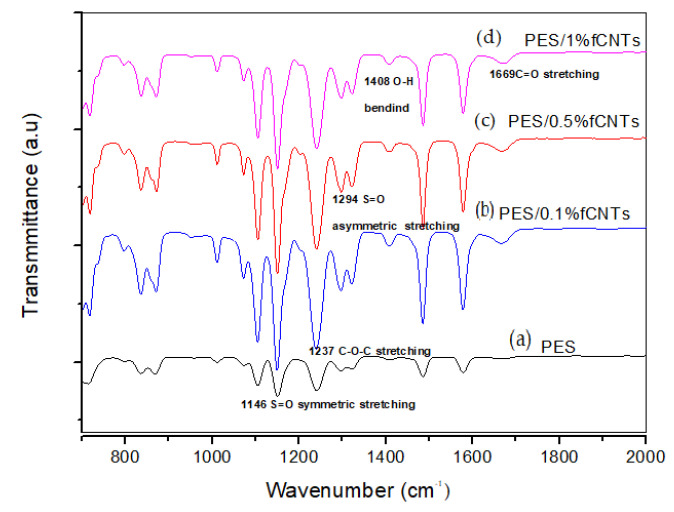
The FTIR spectra of (a) PES membrane; (b) PES/0.1% CNTs membrane; (c) PES/0.5% CNTs membrane; (d) PES/1% CNTs membrane.

Figure 2 displays the FTIR spectra of the PES membrane and the PES/fCNTs membranes in several percentages of fCNTs (0.1, 0.5 and 1%). The FTIR spectrum of the blend membrane showed new peaks at 1669 cm^−1^ and 1408 cm^−1^, corresponding to the carbonyl C=O stretching vibration and the O–H bending vibration of the carboxyl group, respectively [12,27]. These peaks indicate the presence of fCNTs in the surface of the membranes [30]. The bands at 1146 and 1294 cm^−1^ were recognized to be the stretching vibrations of S=O symmetric and S=O asymmetric, respectively. Additionally, the band at 1237 cm^−1^ was attributed to the symmetric C–O–C stretching vibration [12,35].

### 3.2. X-ray Diffraction (XRD)

**Figure 3:**
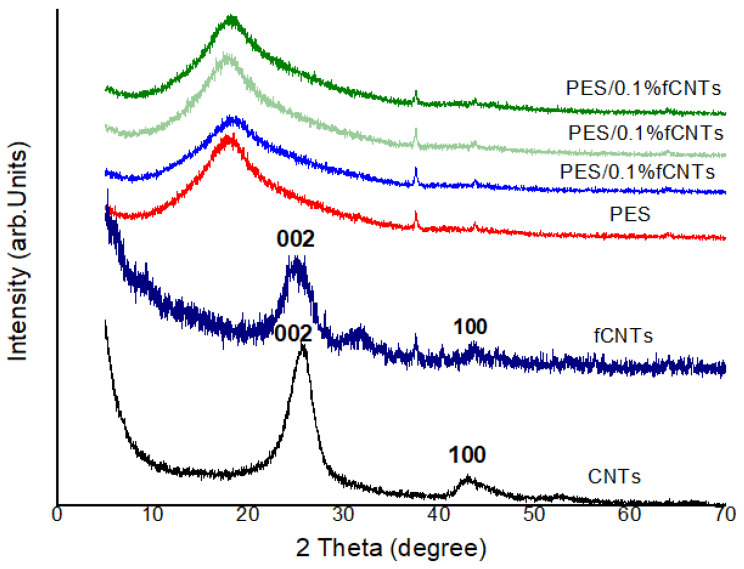
XRD spectra of CNTs and functionalized CNTs.

The X-ray diffraction of the PES membrane and PES/fCNTs with different content is shown in Figure 3. The pattern of the CNTs presented a high intense peak at 26.06° and a low intense peak at 42.94°, corresponding to the (002) and (100) reflections, respectively. Meanwhile, the fCNTs presented a high intense peak at 25.14° with a slight shift toward lower diffraction angles (higher interplanar spacings) which may be attributed to an increase in the sp2 C=C layers spacing [36]. A low intense peak was suppressed because of the presence of a minor phase due to the impurities in the samples during the preparation process. This led to the change in the phase intensities and, thus, a considerable decrease in the second peak intensity of the CNTs [37]. Although there was a drastic decrease in the XRD reflections of the fCNTs’ intensities, deep XRD analysis revealed that the fCNTs structure was still present in the polymer matrix after functionalization [38,39]. As for the membrane of the pure PES XRD pattern, a main, broad amorphous peak at 2θ = 18.4° was observed, which is similar to the reported peak for pure PES [40]. The XRD patterns of the PES/fCNTs displayed a single diffraction peak of PES (2θ = 18.4°) with very small peaks of fCNTs. This indicates that PES diffraction peaks were predominant over those for fCNTs, suggesting the homogeneous dispersion of fCNTs in PES membranes [41]. Moreover, for small amounts of fCNTs, the quite small peak intensities were strongly related to the detection limit of the XRD powder diffractometer.

After calculating the interplanar spacing of the major diffraction peaks of CNTs (i.e., θ = 26.06°) and fCNTs (θ = 25.14°), we noticed that there was almost no change in the peaks’ positions. This suggests that there was no change in the local crystal structure.

### 3.3. Scanning Electron Microscope (SEM)

**Figure 4:**
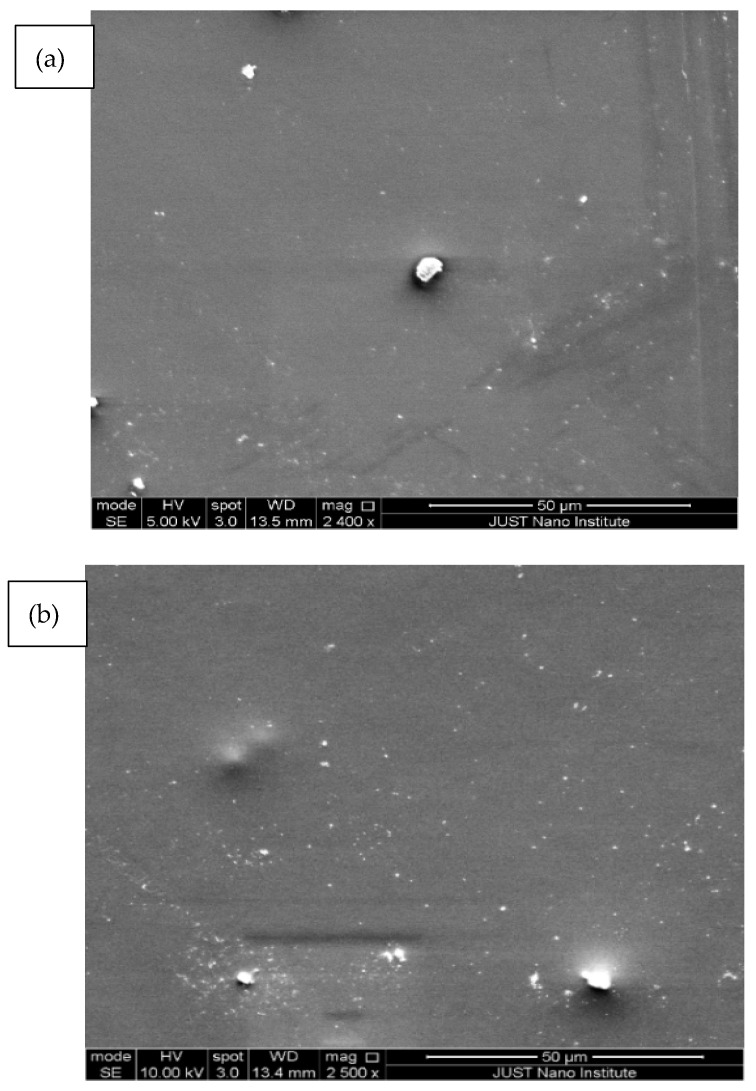

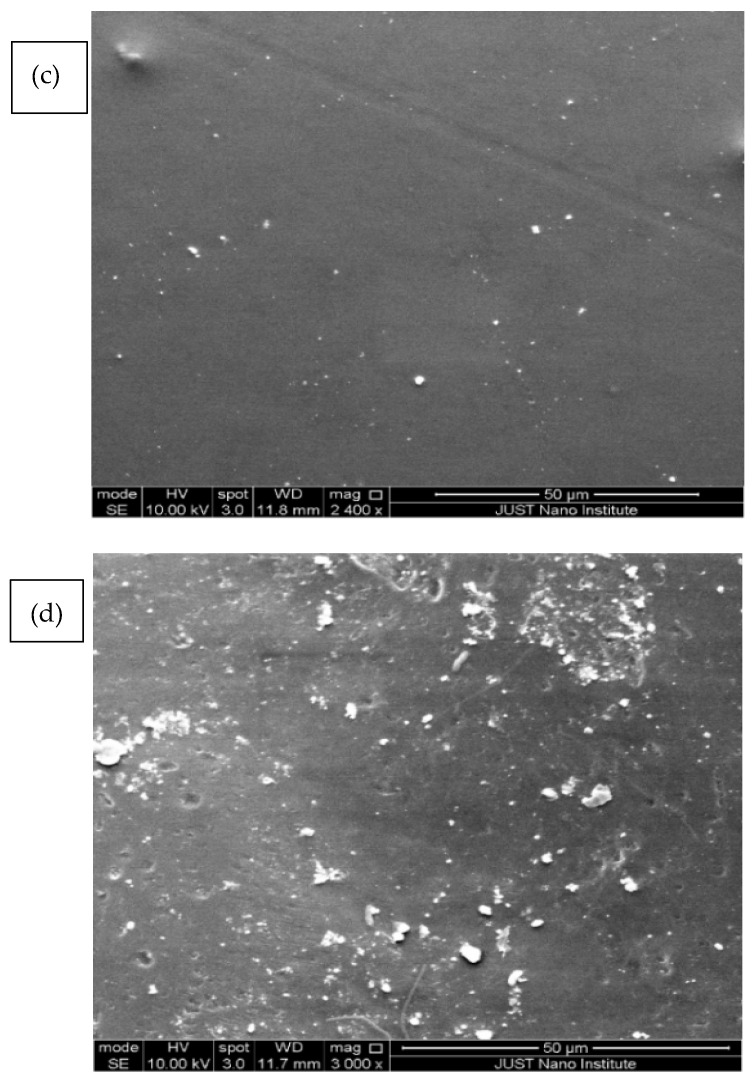
SEM images of the top surfaces of PES/fCNTs membranes for different fCNTs content. (**a**) 0.0 wt.%, (**b**) 0.1 wt.%, (**c**) 0.5 wt.%, (**d**) 1.0 wt.% fCNTs content.

To understand the effect of fCNTs on the PES membrane structure, the morphology of PES/fCNTs membranes was studied by SEM. Figure 4 presents the top surfaces of the PES/fCNTs membranes for different fCNTs content. The images show that the membranes had smooth and dense surfaces due to the fast de-mixing during the phase inversion process. Traces of randomly distributed grain structures were observed on the top surface of the membranes, and it was more obvious for the sample with the highest fCNT content (Figure 4d). The presence of such grain is due to the agglomeration of the CNTs and surface migration during the phase inversion process. The agglomeration of CNTs which arouse from the π–π interactions, in addition to the inter-particle forces between fCNTs, such as van der Waals forces, was more noticeable at a high loading of CNTs [29]. The images clearly indicate that, at a low CNTs loading, the nanotubes were regularly distributed in the polymer matrix, which resulted in a smooth, dense surface; however, as the percentage of CNTs was increased, agglomeration of the nanotubes was observed, which led to increased surface roughness. Similar results were observed by Vatanpur et al. [30] and Qiu et al. [42] 

**Figure 5:**
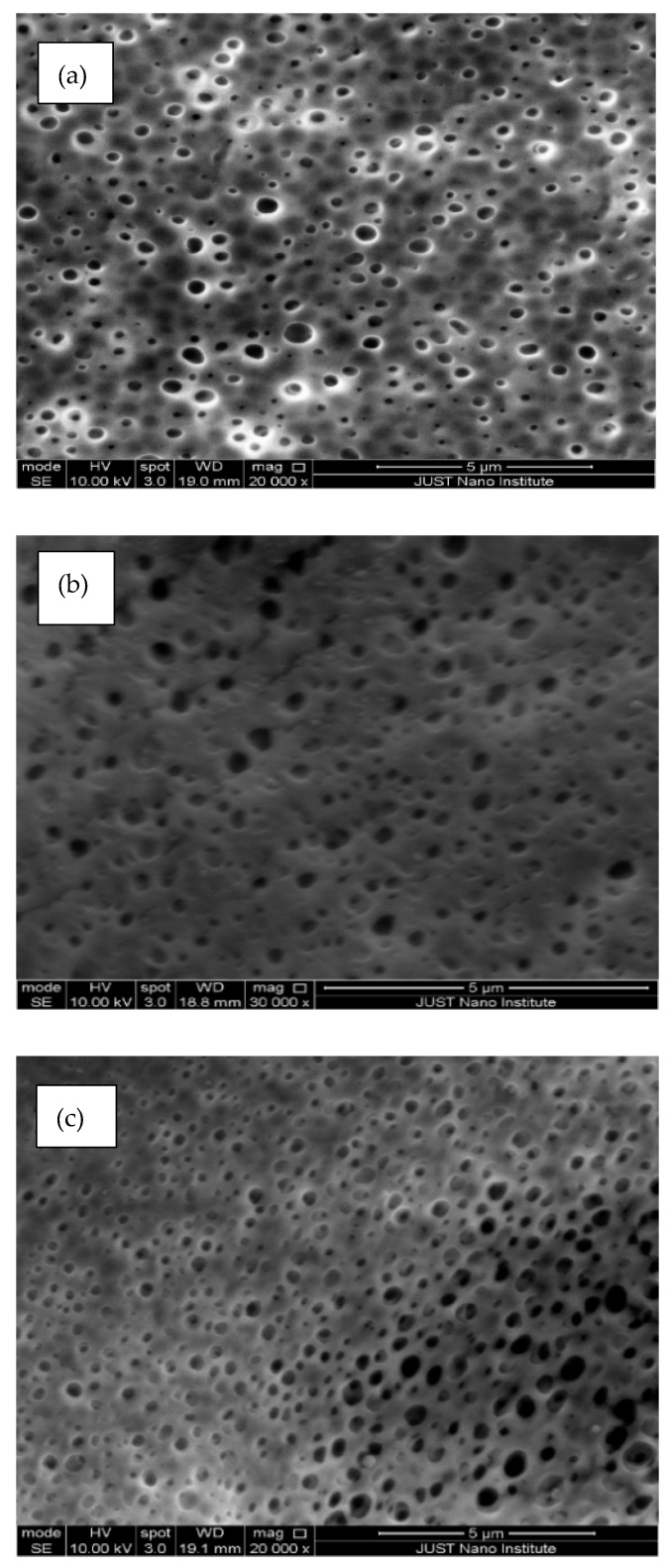

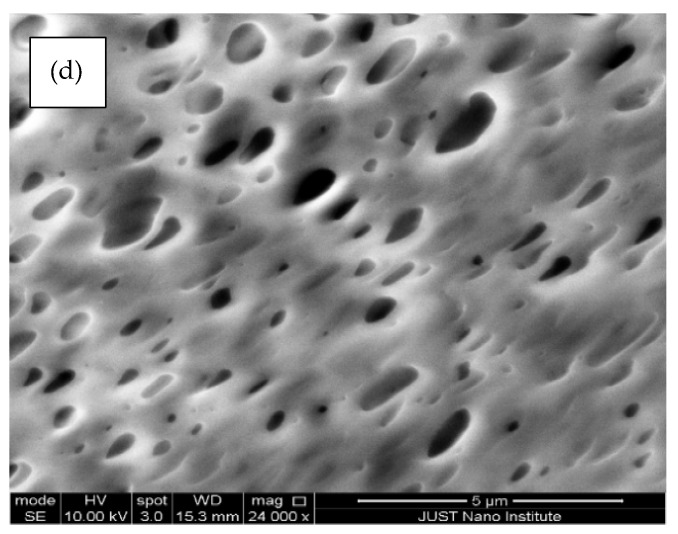
SEM images of the bottom surfaces of PES/fCNTs membranes for different fCNTs content. (**a**) 0.0 wt.%, (**b**) 0.1 wt.%, (**c**) 0.5 wt.%, (**d**) 1.0 wt.% fCNTs content.

Figure 5 shows the microstructure of the membrane’s bottom surfaces for various fCNTs concentrations. Considerable number of pores with different sizes were formed. The pore density was higher in membranes with fCNTs than for pristine PES. The size of the pores on the bottom surface of each membrane was measured with typical sample position on every SEM image. The SEM images revealed that, as the percentage of fCNTS in membranes increased, the average pore sizes increased from about 0.3 µm in the PES membrane to 0.5 µm in the PES/fCNTs membrane (with 1% wt. fCNTs). This result revealed that the formation of pores on the bottom surface of membranes increased by adding fCNTs.

**Figure 6:**
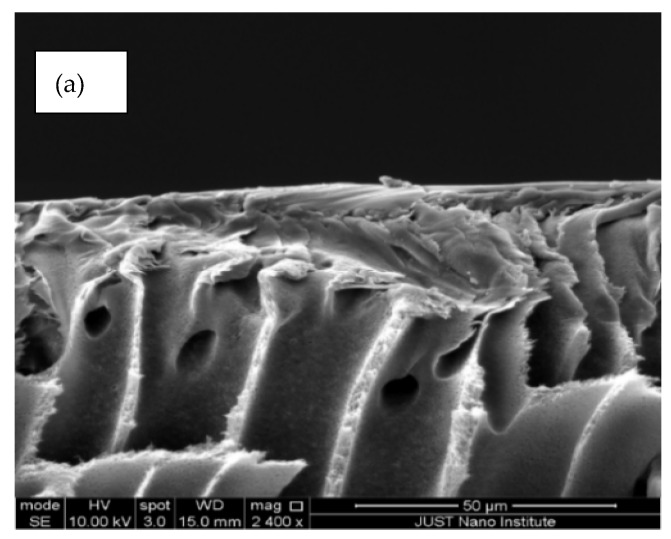

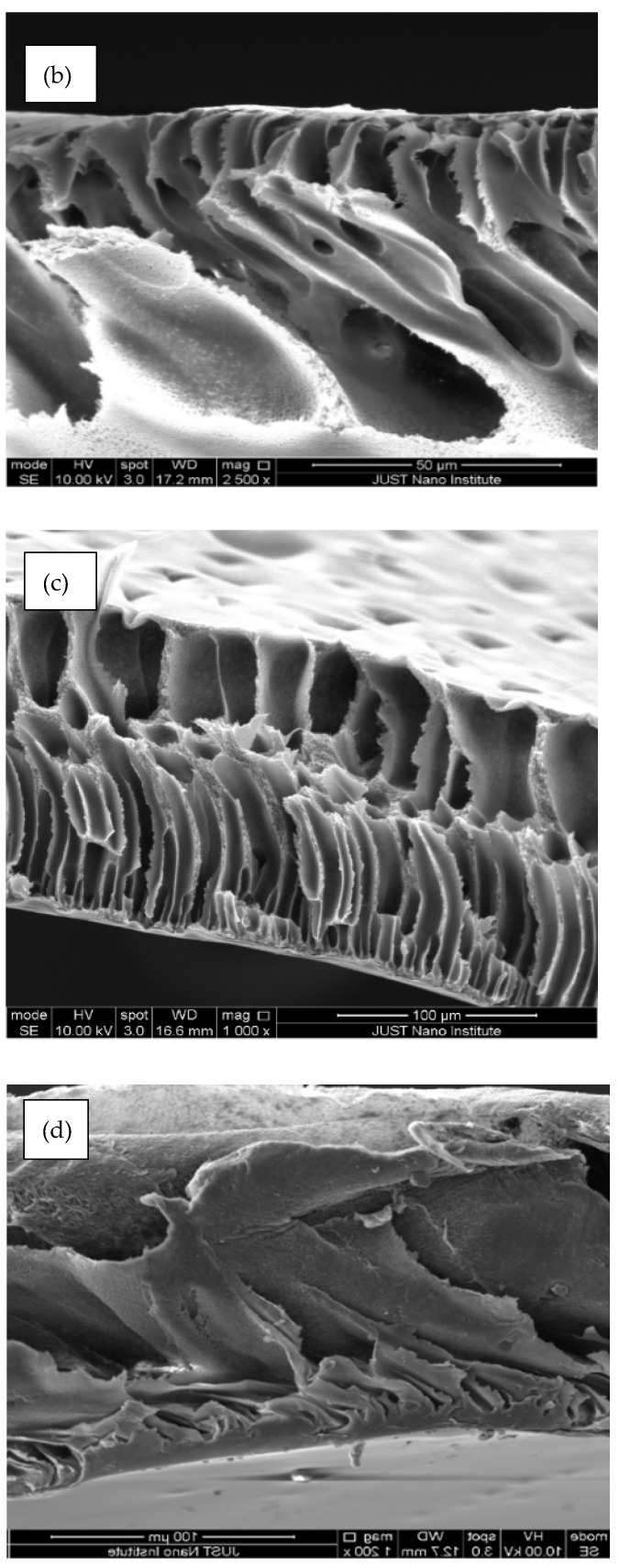
SEM images of cross-sections of PES/fCNTs membranes for different fCNTs content. (**a**) 0.0 wt.%, (**b**) 0.1 wt.%, (**c**) 0.5 wt.%, (**d**) 1.0 wt.% fCNTs content.

Figure 6 illustrates the membranes’ cross-sectional images which are composed of macrovoids at the bottom, asymmetrical, finger-like structures in the middle and a quite dense top layer (of few microns in thickness), whereas, in the membranes with the highest fCNTs content, quite deformed and tilted, finger-like pores were observed. The formed macrovoids at the bottom layer were thick. This result indicates that the addition of the hydrophilic fCNTs in the casting solution can lead in the phase inversion process to fast exchange between solvent and non-solvent [43]. Moreover, the presented bottom images of membranes and the size of the membrane pores for the membranes were apparently greater than that of the PES membranes. These results suggest well-distributed CNTs in the membrane [44]. Our results agree with Wang et al. [28], and Vatanpour et al. [30]; they reported a dense top layer, a porous sub-layer with finger-like and macropores at the bottom of the PES/fCNTs membranes.

### 3.4. Contact Angle Measurement

**Figure 7:**
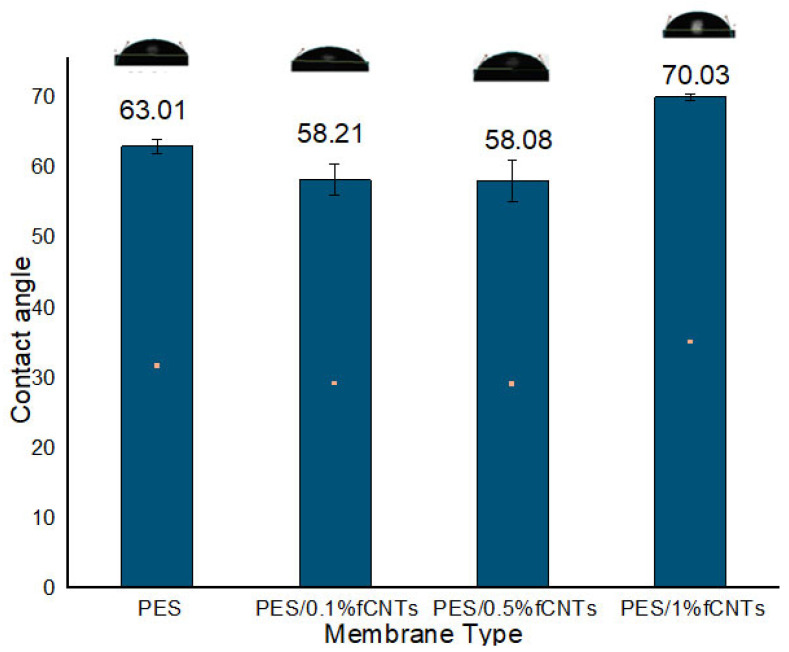
Average contact angles of the PES/fCNTs membrane surfaces.

The contact angle method was used to determine the hydrophilicity of the PES/fCNTs membranes’ surfaces. The sessile drop method was used to determine the contact angles of the membranes [30]. As presented in Figure 7, the contact angles of the blend membranes decreased when adding fCNTs, which led to an enhancement of the membrane hydrophilicity. Meanwhile, when the fCNT amount reached 1%, the hydrophilicity did not enhance; that could be ascribed to the irregular sitting of the fCNTs in the structure of membrane at 1% fCNT content [27,42].

As shown in Figure 7, the PES membrane, in the absence of fCNT, had a contact angle of 63.01°, and an enhancement in hydrophilicity was observed as the amount of fCNT increased to 0.5% fCNTs with lower water contact angles of 58.08° for the PES/0.5% fCNT membranes [30]. This could be due to the spontaneous migration of hydrophilic fCNTs to the membrane/water interface during the phase inversion process to reduce the interface energy [45].

### 3.5. Porosity

Table 1 displays the porosity of synthesized membranes with 0, 0.1, 0.5 and 1 wt.% fCNTs. According to the results, the porosity of all membranes was higher than the pure PES membrane. The results indicated an increase in porosity as the number of fCNTs increased. These results agree well with the SEM results in which the number of fCNTs in the membrane enhanced the membrane’s porosity.

### 3.6. Membrane Performance Testing

#### 3.6.1. Pure Water Flux for Membranes

**Figure 8:**
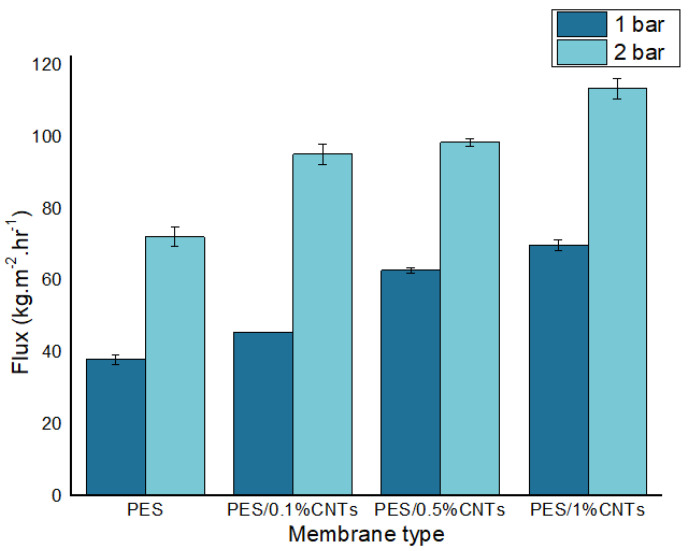
Pure water flux of synthesized membranes at operating pressure of 1 and 2 bar. Samples were run in triplicate.

Figure 8 displays the pure water flux of the synthesized membranes (0, 0.1, 0.5 and 1 wt.% fCNTs) at working pressures of 1 and 2 bars. It was detected that the pure water flux of PES/fCNTs membranes was more than the PES membrane. The flux of the membranes was influenced by the structural features and contact angle as a function of hydrophilicity [43]. The hydrophilic groups of the fCNTs surface improved the hydrophilicity of the membrane surface (see Figure 7). This increase in hydrophilicity led to an increase in water flux which agreed with our contact angle results. By careful examining the SEM image (Figure 6d), it can be noticed that the membrane with the highest CNT content (1%) had a different internal and surface structure than other membranes where the porous structure seems to have collapsed. This can lead to loss of mechanical and structural integrity of the membrane which leads to loss of functionality. The same observation can be made from Figure 5d, showing large and irregular pores. This is most likely due to agglomeration of the CNTs and/or reduced distribution of the CNTs inside the membrane which is expected in the case of nanomaterial distribution in a polymer matrix. This explains the result of the high flux due to larger pores but loss of functionality due to the loss of mechanical and structural integrity of the membrane.

According to Figure 8, an increase in CNTs content in PES membranes led to higher pure water flux. Therefore, PES/1% fCNTs membrane indicated the highest water flux of 69.71 ± 1.38 (kgh^−1^m^−2^) at 1 bar.

**Figure 9:**
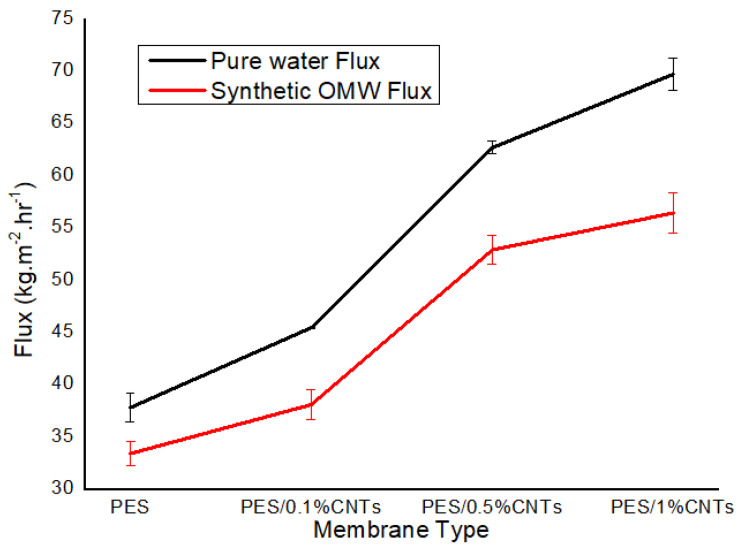
Pure water flux vs. synthetic OMW flux for synthesized membranes at operating pressure of 1 bar, time 30 min. Samples were run in triplicate.

Figure 9 demonstrates a comparison between the permeability flux of pure water and synthetic OMW. It shows that synthetic OMW has less permeate flux because of the presence of the oxygen-containing functional groups on the surface of fCNTs [46]. Additionally, it could be attributed to membrane surface fouling due to other components’ deposition in the synthetic OMW, which is in line with results obtained by Daramola et al. [14].

#### 3.6.2. Rejection Percentages

**Figure 10:**
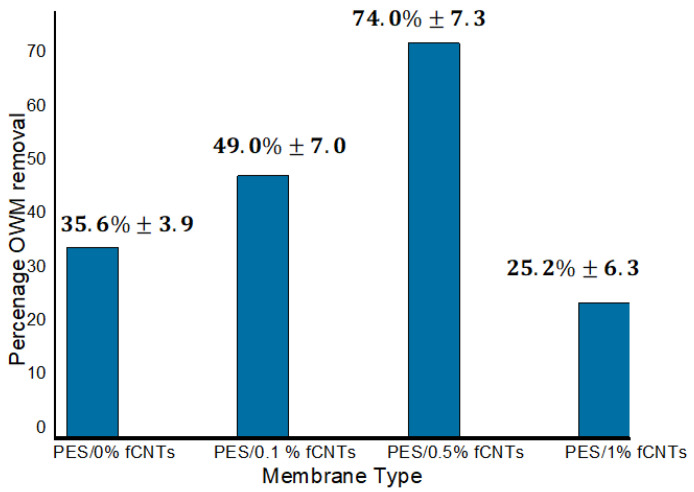
TPC rejection percentages for PES/fCNTs membranes at different fCNTs percentage. Experimental conditions: pressure, 1 bar; concentration of phenol, 74 ppm. Samples were run in triplicate.

Figure 10 shows the removal of total phenolic content (TPC) for PES/fCNTs membranes compared with pure PES membrane. As seen in the figure, the increase of fCNTs concentration from 0 to 0.5 wt.% led to an increase in the TPC rejection to 74% for the PES/0.5% fCNTs membrane. This can be attributed to well-distributed fCNTs in the membrane which led to higher TPC rejection. The observed SEM results indicated more significant dispersion for fCNTs at 0.5 wt.% (Figure 5c) compared to fCNTs at 0.1 wt.% (Figure 5b), thus enhancing the active sites in the membrane surface, which led to higher rejection.

On the other hand, the rejection decreased for the membrane with 1 wt.% of fCNTs to 25%. The rejection of the phenolic compounds by the membrane was attributed to both adsorption and size exclusion mechanisms. At low CNTs percentages, the adsorption mechanism was dominant due to the presence of the CNTs which have high affinity toward phenolic compounds; however, at higher CNTs, the size exclusion mechanism became more dominant due to the increased pore size, as was shown in the SEM analysis above. This may increase the penetration of the phenolic compounds through the membrane (i.e., pores larger that TCP). Combined with the loss of active sites due to agglomeration of the CNTs, the overall rejection of the membrane was decreased at 1 wt.% of fCNTs. These results agree well with Rameetse et al. [47] findings for PSF/PES/CNTs, in which they studied the effect of pure CNTs and fCNTs on benzene and phenol rejection. The percentage of rejection in their membranes was found to decrease as the concentration of CNTs increased.

## 4. Conclusions

The PES/fCNTs membranes were prepared by the phase inversion method. The surface of the CNTs were modified by H_2_SO_4_:HNO_3_ to increase the dispersion of CNTs in organic solvent.

The prepared membranes were characterized by scanning electron microscopy (SEM), Fourier-transform infrared spectroscopy (FTIR) and contact angle. Meanwhile, the effect of CNTs on membranes was remarkable, as shown in SEM images. Adding the fCNT into membranes increased the finger-like pores in the sub-layer, resulting in higher porosity of the membranes’ sub-layer. Significantly, the contact angles of the membranes decreased as the content of fCNTs increased, resulting in an enhancement in the membrane hydrophilicity.

Notably, the amount of fCNTs in membrane was the most significant factor affecting the membrane’s hydrophilicity, morphology and water flux. The PES/fCNTs membranes with 1 wt.% of CNTs presented the highest water flux of 69.71 (kgh^−1^m^−2^)at 1 bar and the lowest water flux of 37.8 (kgh^−1^m^−2^) at 1 bar for PES with 0% fCNTs.

TPC removal increased with increasing the amount of fCNTs percentage from 0 to 0.5 wt.%, whereas at 0.5 wt.% the rejection reached 74% and decreased for samples prepared with 1 wt.% of fCNTs.

The results of this work indicated the potential use of PES/fCNTs membranes in removing total phenol compounds from OMW if prepared at optimum concentration and functionalization of CNTs. More examinations are still required to enhance the CNTs surface with other functional groups and study their performance and fouling behavior under different conditions.

## Figures and Tables

**Table 1:** Porosity of the membranes.

Membrane Type	Porosity (%)
PES	82.78 ± 0.03
PES/0.1% fCNTs	84.52 ± 0.02
PES/0.5% fCNTs	86.89 ± 0.01
PES/1% fCNTs	87.34 ± 0.02

## Data Availability

Data from this study can be made available upon request.
